# Effect of Washing on the Electrochemical Performance of a Three-Dimensional Current Collector for Energy Storage Applications

**DOI:** 10.3390/nano11061596

**Published:** 2021-06-17

**Authors:** Sajid Ali Ansari, Nazish Parveen, Mohd Al Saleh Al-Othoum, Mohammad Omaish Ansari

**Affiliations:** 1 Department of Physics, College of Science, King Faisal University, P.O. Box 400, Hofuf 31982, Saudi Arabia; malothoum@kfu.edu.sa; 2 Department of Chemistry, College of Science, King Faisal University, P.O. Box 380, Hofuf 31982, Saudi Arabia; nislam@kfu.edu.sa; 3 Center of Nanotechnology, King Abdulaziz University, Jeddah 21589, Saudi Arabia; moansari@kau.edu.sa

**Keywords:** current collector, energy storage, electrochemical performance

## Abstract

The development of efficient materials for energy storage applications has attracted considerable attention, especially for supercapacitors and batteries that are the most promising and important power sources in everyday life. For this purpose, a suitable and efficient current collector must be determined and its behavior with respect to various solvents when it is used as an electrode material for energy storage applications should be understood. In this work, we studied the effect of washing three-dimensional nickel foam using different concentrations of hydrochloric acid and ethanol on the surface characteristics, electrochemical behavior, and storage performance of the foam. Additionally, we observed the different types of acidic treatments that improved the electrochemical and storage performances of the three-dimensional nickel foam. The surface characterization results show that acidic conditions with a concentration of 3M changes the surface morphology from a flat/hill-like structure to a nanosheet/nanoflake-like structure without any further treatment. This structure provides a nano-channel and a large number of surface charges during the electrochemical reaction. The results of this study show that pretreatment of 3D-NF is highly important and recommended. The present work also contributes to the knowledgebase on pretreatment of 3D-NF and its optimization.

## 1. Introduction

Energy storage devices, especially supercapacitors (SCs) and batteries, are the main chemical-based modern sources of energy that are widely used for various devices in everyday life such as vehicles and toys [1,2,3,4,5]. However, the increasing demand for these devices is of serious concern because of their dependency on energy, especially energy from fossil fuels, which leads to environmental issues in terms of pollution. To avoid these problems, researchers have been investigating alternatives to fossil fuels, such as electrochemical supercapacitors, fuel cells, and batteries [1,2,3,4,5,6,7,8,9]. Among all energy conversion systems, supercapacitors have received considerable attention, and a number of energy storage devices have been developed and commercialized. Apart from these successes, SCs have also been considered as efficient next-generation energy storage devices owing to their rapid charging/discharging process, long life stability, high safety, and rapid energy delivery process. At the same time, the key obstacle of supercapacitor regarding low energy density has been known as a foremost shortcoming in the continuance of supercapacitor technologies [10]. Based on the charge storage mechanism, supercapacitors can be classified into three types: (i) electrochemical double layer capacitors store charges via the electrochemical double layer formation/non-Faradaic mechanism/electrostatically, (ii) pseudocapacitor store charges via reduction-oxidation/faradaic process, and (iii) asymmetric supercapacitors store the charge both by Faradaic and non-Faradic process. The energy density and power density are the important factors to evaluate the performance of supercapacitors. As can be seen, the energy density of capacitors is directly dependent on the specific capacitance and the voltage, so to increase energy density, either specific capacitance or potential or both of these quantities should be increased. This can be achieved by using electrode materials with high specific surface area, suitable electrolyte, and optimization of the structure of the integrated systems.

In addition to these characteristics, the SC is made of an anode and a cathode, which are divided by an appropriate separator. The anode/cathode can be developed by either coating active materials onto the conductive current collector or grown of the active materials directly on the surface of the current collector. However, the SC performance depends on the current collector behavior of the electrode (i.e., the active material), including electrical conductivity, surface area, porosity, electrochemical activity, and morphological geometries. Therefore, the selection of an appropriate current collector for effective electrode development is highly important as it acts as a bridge between the active materials and the outer terminal during the energy storage process. There are mainly two chemical etching and coating processes that have been applied for treating the current collector. The chemical etching/treatment process efficiently roughens the exterior/surface of the current collectors, which is advantageous for enhancing adhesion and interfacial conductivity between electrodes and current collectors as well as ions transportation through electrode/electrolyte. Moreover, coating is considered a significant route to alter the surface material to realize improved performance. However, the chemical etching process might also bring a risk of contamination and reduce the mechanical strength of the current collectors [11].

Therefore, various types of current collectors have been used, such as two-dimensional carbon paper, metal-based meshes, metal-based wires, steel mesh, graphite rods, and three-dimensional nickel foam. Three-dimensional nickel foam (3D-NF) has attracted considerable attention for the development of energy storage devices owing to its high surface area, excellent electrical conductivity, controllable pore size, low cost, and highly stable three-dimensional structure. The large surface area and three-dimensional structure are helpful for interaction with the electrolyte during the electrochemical process; the porosity reduces the path length of the ionic diffusion, which enhances the overall performance of the electrodes. In addition, the low toxicity, high mechanical strength, inertness, and corrosion stability behavior in alkaline solution make it a suitable current collector for energy storage devices. Therefore, 3D current collectors, and especially 3D-NF, have been used in various energy storage devices; these collectors improve the overall specific capacitance performance of the electrodes. Apart from the ideal characteristics of 3D-NF as a current collector, its proper pretreatment is still not very clear. The foam needs to be pretreated because the surface is covered with a thin layer of nickel oxide. If the 3D-NF surface is not pretreated properly, the nickel oxide can be converted into electrochemically active nickel hydroxide; this contributes to the additional capacitance value of the electrode. The thin layer on the surface of the 3D-NF and its activity in the presence of the electrolyte could be considered as a significant problem. A few studies have examined pretreatment or washing of 3D-NF, but they have not directly focused on it. For example, Grden et al. studied the surface science and electrochemical performance of nickel foam [12], whereas Su et al. reported the capacitance performance of a LiNi_0.90_Co_0.06_Mn_0.04_O_2_ material surface washed with a low concentration of boric acid [13]. Similarly, Salleh et al. reported the comparative performance of nickel foam and nickel mesh [14]. Kovalenko et al. studied the activation process of nickel foam and its effect on the electrochemical performance, which can improve the electrochemical supercapacitive performance of the device [15].

In this study, it is shown that pretreatment of 3D-NF is highly important and recommended. The present work also contributes to the knowledgebase on pretreatment of 3D-NF and its optimization. In our work, 3D-NF was washed with different concentrations of acid and alcohol and further effects on the surface behavior were studied in detail using scanning electron microscopy. The electrochemical performances of untreated and pretreated 3D-NF were examined by cyclic voltammetry and galvanostatic charge/discharge techniques in a three-electrode assembly cell. The obtained electrochemical results clearly showed that washing 3D-NF with different concentrations of acid and alcohol significantly affected the electrochemical performance of the 3D-NF.

## 2. Materials and Methods

### 2.1. Materials

Hydrochloric acid, ethyl alcohol, and potassium hydroxide were purchased from Sigma-Aldrich (St. Louis, MO, USA). Nickel foam (>99.99% purity) was obtained from MTI Corporation (Richmond, CA, USA).

### 2.2. Washing and Pretreatment of 3D-NF

Several pieces of 3D-NF of size 3 × 1 cm were cut from commercially available 3D-NF sheet. A few pieces of the 3D-NF were immersed in hydrochloric acid (1 and 3M), and further sonicated in an ultrasonication bath for 15 min. After completing the sonication process, the washed 3D-NF were rinsed with deionized water and ethanol and dried in an oven. For the comparison study, a few pieces of the 3D-NF were immersed in ethanol, and further sonicated in an ultrasonication bath for 15 min. The washed 3D-NF pieces were dried in an oven for further study.

In this study, commercial 3D-NF is abbreviated as 3D-NF-C, whereas the 3D-NF that are washed with ethanol, 1M HCl, and 3M HCl are abbreviated as 3D-NF-etOH, 3D-NF-1MH, and 3D-NF-3MH, respectively. 

### 2.3. Methods

Surface changes were observed using a field-emission scanning electron microscope (SEM; S4800, Hitachi, Tokyo, Japan). 

### 2.4. Electrochemical Measurements

A three-electrode assembly setup (3D-NF = working electrode, Ag/AgCl = reference electrode, Pt counter electrode) attached to VersaSTAT3 (Princeton, Research, Princeton, NJ, USA) was used to evaluate the electrochemical performance of the washed and untreated 3D-NF. 

## 3. Results and Discussion

### Scanning Electron Microscopy (SEM) Analysis

**Figure 1:**
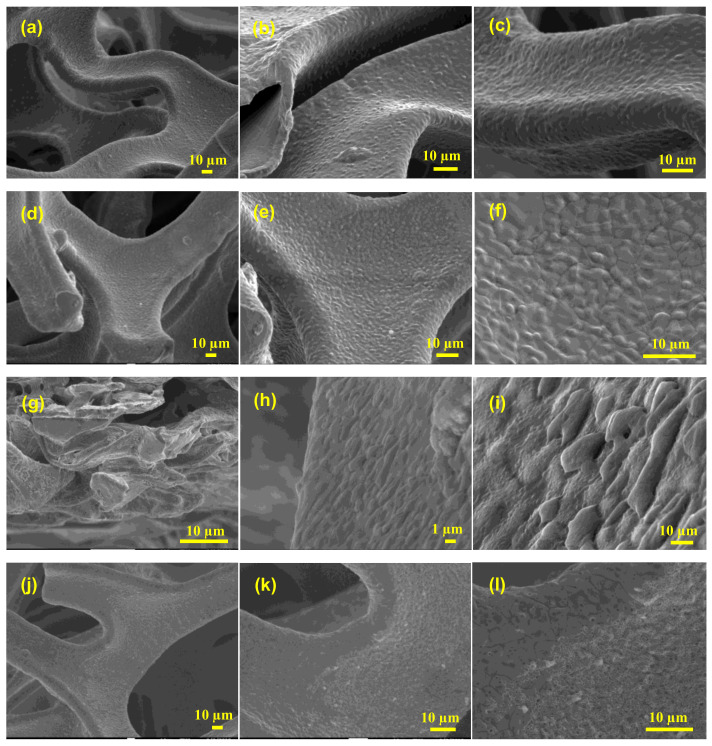
(**a**–**c**) SEM images of commercial Ni-foam; (**d**–**f**) SEM images of Ni-foam washed with ethanol; (**g**–**i**) SEM images of Ni-foam washed with 1M HCl solution; and (**j**–**l**) SEM images of Ni-foam washed with 3M HCl solution at different magnifications.

**Figure 2:**
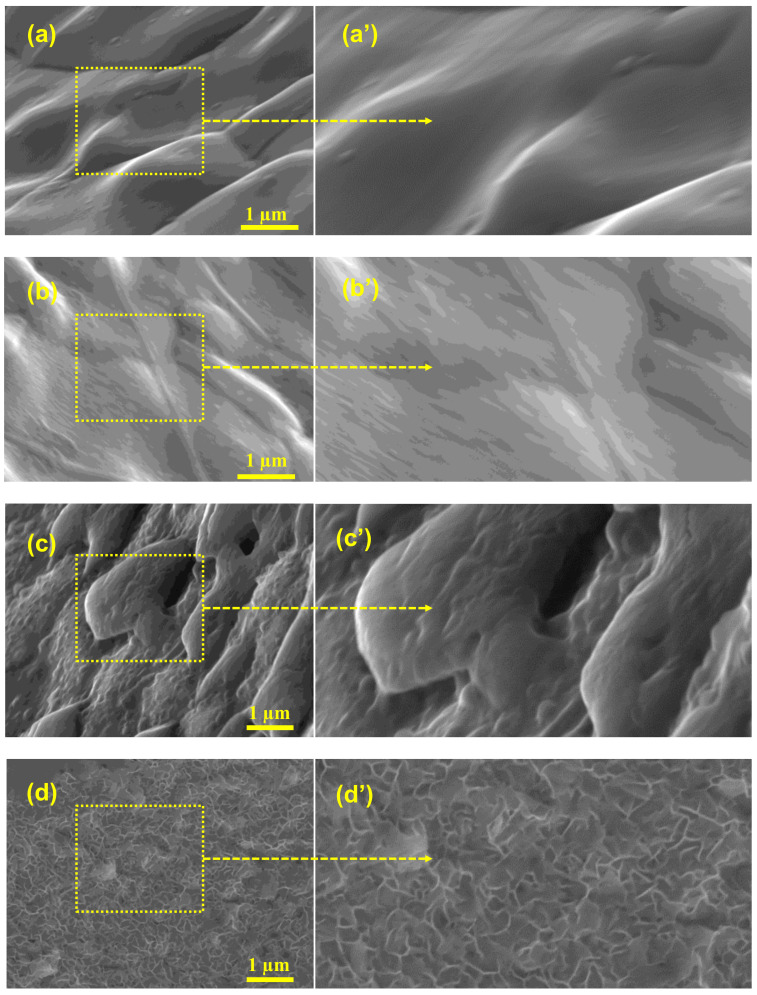
(**a**,**a’**) Closed inspection analysis of the SEM image of the Ni-foam; (**b**,**b’**) closed inspection analysis of the SEM image of the Ni-foam washed with ethanol; (**c**,**c’**) closed inspection analysis of the SEM image of the Ni-foam washed with 1M HCl solution; and (**d**,**d’**) closed inspection analysis of the SEM image of the Ni-foam washed with 3M HCl solution.

Commercially accessible nickel foam was used as an inert three-dimensional binder-free substrate and/or a current collector; this foam possesses the capability to include various materials into its 3D structure, and allows a larger interfacial contact than two-dimensional substrates. The unique construction of nickel foam allows it to possess a large surface area, high microporosity and mesoporosity, high intrinsic electrical conductivity, and exceptional chemical and mechanical stability in a wide range of liquid electrolytes. To investigate the effects of washing on the surface/electrochemical behavior of the bare nickel foam, three different washing solvents were selected. The surface morphology and microstructure of the bare NF, 3D-NF-etOH, 3D-NF-1MH, and 3D-NF-3MH samples at different magnifications are presented in Figure 1 and Figure 2. It can be seen that the commercial NF possesses a smooth surface in three dimensions with large pores of size several tens of micrometers, as shown in Figure 1a–c. The NF that is washed with EtOH is shown in Figure 1d–f, where a loose and porous morphology is combined with the presence of etch pits. After acidic treatment with 1M HCl, the nickel foam surface seemed to become rougher; after washing with a higher acidic concentration, the entire NF had a visible corrosive surface with porosity throughout the substrate, as shown in Figure 1g–i. An in-depth examination of the surface morphology can be obtained by high-magnification SEM analysis, as shown in Figure 2. A close inspection showed that there were significant uniform wrinkles on the surface of the NF (Figure 2a,a’). According to morphological analysis, bare Ni foam has a less porous structure compared to NF washed with ethanol NF, which has a planar surface with a rough morphology of hill-like structures (Figure 2b,b’). Washing with hydrochloric acid could have a greater effect on the NF surface; this might increase the specific surface area, which would enhance the specific capacity. As a result, low-magnification SEM analysis of surface washing with 1M HCl was performed. It could be observed that in addition to etch pits, hill-like nanosheets (as shown in Figure 2c,c’ started to grow on the surface of the NF scaffold. When the concentration was increased to 3M, the coverage area of the nanosheets/nanoflakes also increased and completely and uniformly covered the surface of the face of the 3D porous substrates (Figure 2d,d’). The grown nanoflakes lay perpendicular to the NF surface; they were interconnected with each other, and developed well-organized nanosheet/nanoflake arrangements with a considerably open and porous assembly [16]. These open nanosheet surfaces can be highly accessible to the electrolyte when they are cast-off as electrode materials for SCs. Several pore formations exist in the nickel scaffold with a cross-linked construction; these irregular porous network frameworks would significantly increase the contact area and decrease diffusion and movement pathways of electrolytic ions, resulting in improved electrochemical performance.

After confirming the washing effect of several solvents on the nickel foam framework, it could be concluded that the modified nickel framework could substantially affect the configuration and electrochemical characteristics of the active materials that are directly prepared on the nickel scaffold. To shed light on the effects of washing on the electrochemical performance, nickel foam was directly modified with various solvents. The electrochemical performance of any material is affected by its surface morphology and structure. Generally, the microstructure of an electrode surface provides a large surface area of the electroactive material. This type of surface allows for better intercalation of electric charges in the electrode material. Different solvents used for washing alter the surface morphology and electrochemical performance. To confirm this, 3D-NF-C, 3D-NF-etOH, 3D-NF-1MH, and 3D-NF-3MH electrodes were employed as electrodes for SCs.

**Figure 3:**
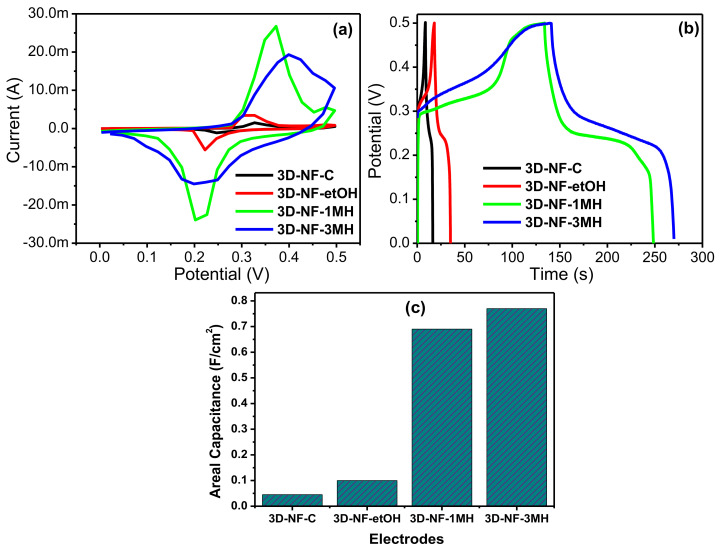
(**a**) Comparative CV curves; (**b**) comparative CD curves, (**c**) areal capacitances of the 3D-NF-C, 3D-NF-etOH, 3D-NF-1MH, and 3D-NF-3MH electrodes.

**Figure 4:**
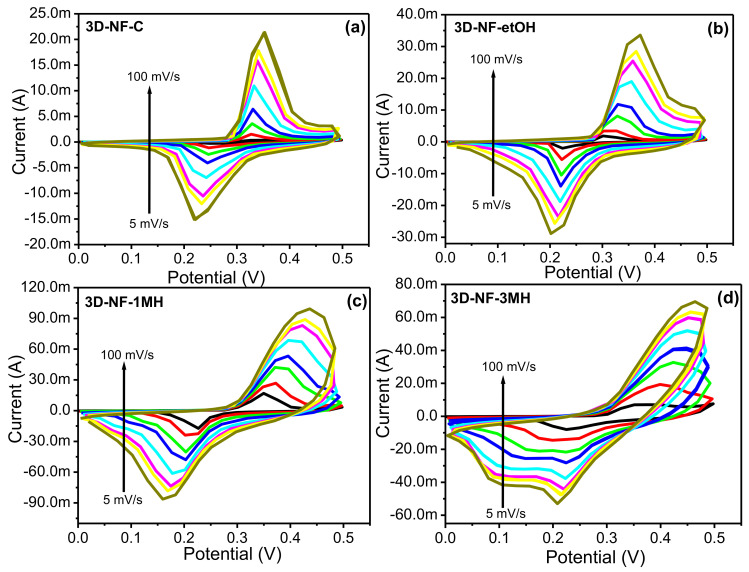
CV graph of the (**a**) 3D-NF-C; (**b**) 3D-NF-etOH; (**c**) 3D-NF-1MH; and (**d**) 3D-NF-3MH electrodes at different scan rates.

**Figure 5:**
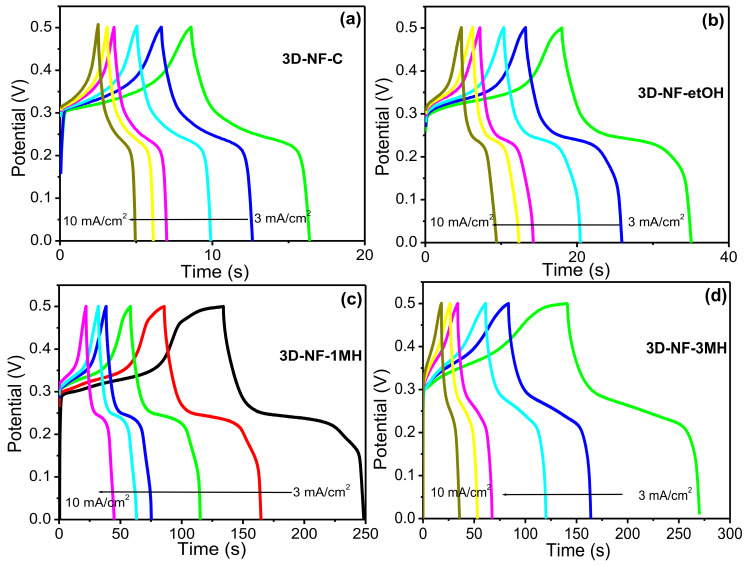
CD graph of the (**a**) 3D-NF-C; (**b**) 3D-NF-etOH; (**c**) 3D-NF-1MH; and (**d**) 3D-NF-3MH electrodes at different current densities.

**Figure 6:**
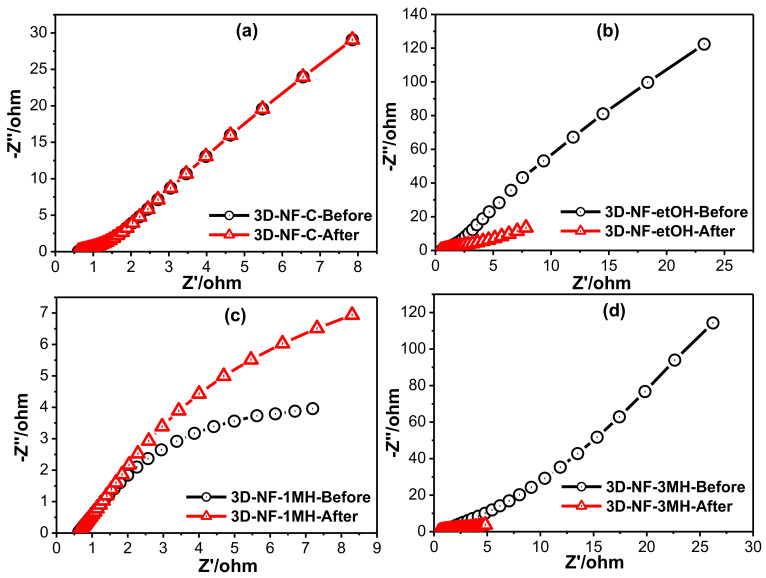
Electrochemical impedance spectra of the (**a**) 3D-NF-C; (**b**) 3D-NF-etOH; (**c**) 3D-NF-1MH; (**d**) 3D-NF-3MH before and after electrochemical measurements.

Electrochemical measurements were performed using cyclic voltammetry (CV) and galvanostatic charge/discharge measurements. Electrochemical impedance measurements (Figure 3, Figure 4, Figure 5 and Figure 6) using a three-electrode system in a 2M KOH electrolyte were performed to compare the electrochemical behavior of the washed foams to that of commercial Ni foam, and to choose the optimal solvent for high performance conditions for electrode preparation. The CV profiles of the different electrodes are presented in Figure 3a. The CV curves of the 3D-NF-C, 3D-NF-etOH, 3D-NF-1MH, and 3D-NF-3MH electrodes are dissimilar from the typical rectangular nature that results from the electrical double layer capacitance; this suggests that pseudocapacitance charging/discharging dominates. A few oxidation and reduction peaks, such as the anodic peak (positive current density) and cathodic peak (negative current density) can be clearly observed in all CV profiles; this suggests that charge storage occurred due to the redox reaction rather than the EDLC produced through proton intercalation and de-intercalation [17]. The 3D-NF-3MH electrode possessed greater currents and a higher area under the CV compared with the 3D-NF-etOH, 3D-NF-1MH, and 3D-NF-3MH electrodes. This clearly reveals that the charge storage behavior of the electrode is primarily attributed to the pseudocapacitance, which is followed by the Faradaic processes of the high surface area of the porous nanosheet-grown morphology. The redox process takes place for all electrodes. A close inspection of the CV profiles of the 3D-NF-3MH electrodes shows that the nature of the CV curve has a rectangular shape with clear redox peaks within the highest CV area; this suggests that pseudocapacitance occurs in the electrochemical behavior of this electrode [15]. This may be due to the formation of nanosheet-like structures on the nickel framework. Once the CV analysis confirmed the improved capacitance of the electrode along with the electrochemical storage mechanism (pseudocapacitance), we further studied the galvanostatic charge/discharge performance of the washed electrodes at a current density of 3 mA/cm^2^ in the potential range 0–0.45 V. 

A comparison of the CD analysis of commercial NF and 3D-NF-etOH, 3D-NF-1MH, and 3D-NF-3MH electrodes is provided in Figure 3b. Similar to the observation drawn from the CV results of these different electrodes, the charge/discharge time for the nickel foam electrode prepared by washing with 3M HCl increased significantly compared to that of the rest of the electrodes. Therefore, the higher charge and discharge times for the 3D-NF-3MH electrode confirm its enhanced supercapacitance performance. The improved performance of the 3D-NF-3MH electrode could be attributed to the formation of distinctive nanosheets on the nickel frameworks. The 3D nickel foam delivers improved electric conductivity and a larger surface area and can encourage/influence the redox reactions; the nanosheets also might add additional redox processes with the electrolyte upon electrochemical progression, thereby increasing the total capacity to some extent [18]. Furthermore, considering the ideal linear charge/discharge shapes that mainly come from the electrical double-layer capacitance, the distinctive plateau curve that was noticed in the CD profile verified once again the leading Faradaic reaction of these electrodes. Therefore, the detected plateaus in the charge/discharge profiles are similar to the CV reflections. In conclusion, Figure 3b provides a direct comparison between the supercapacitance performances of the commercial NF electrodes with that of the NF electrodes washed with different solvents.

Evidently, at the same scan rate of 5 mV/s, the CV curve of the 3D-NF-3MH electrode indicates a significantly higher redox current, and a considerably larger area under the CV compared to other electrodes; this indicates the enhanced electrochemical capacitance performance of the washed 3D-NF. Similarly, from the charge/discharge profile, it is clear that the 3D-NF-3MH electrode shows a higher charge/discharge time than the other electrodes at the same current density (3 mA/cm^2^). The calculated areal capacitances of the commercial NF and 3D-NF-etOH, 3D-NF-1MH, and 3D-NF-3MH electrodes at 3 mA/cm^2^ were approximately 0.045, 0.1, 0.69, and 0.77 F/cm^2^, respectively.

This set of analyses clearly shows that the modified surface nickel foam with 3M HCl has improved capacitance compared to commercial nickel foam and other modified surfaces. These improved charge storage abilities might be due to the formation of porous or void nanosheets, which can improve the concentration of active sites or interactions of the electrode/electrolyte surface. This improved concentration results in low interior resistance and shortening as well as easy migration of the electrolyte ions for improved electrochemical reactions, specific capacitance, energy, and power density.

To further investigate the change or washing effect on NF during the electrochemical process, CV and CD tests were performed at various scan rates and current densities (Figure 4 and Figure 5). The representative CV and CD curves of bare NF, 3D-NF-etOH, 3D-NF-1MH, and 3D-NF-3MH individual electrodes are shown in Figure 4 and Figure 5. The CV curves of 3D-NF-C and 3D-NF-etOH samples have almost similar shapes and show variations of anodic and cathodic sweeps at various scan rates from 5 to 100 mV/s, which is related to the CV profile demonstrated earlier in Figure 3a. It can be clearly seen that both cathodic and anodic sweeps significantly increase linearly with an increasing scan rate; this shows that the redox behavior is mainly due to surface-controlled activity. Figure 5 presents the charge/discharge profiles obtained at various current densities, in which the distinctive prospective plateau additionally validates the pseudocapacitive features of the electrodes. Moreover, the charge/discharge time decreased with an increasing current during the electrode performance in the three-electrode system. This feature suggests that the ideal Faradaic redox process occurs dominantly during the electrochemical process; this can be depicted as Ni(II) ⇔ Ni(III) + e, suggesting a conventional pseudocapacitance mechanism. 

Furthermore, the profile of the CV curves barely varied at various scan rates, indicating the excellent reversibility of the anodic and cathodic sweeps. The CV curves have relatively smaller slopes at lower scan rates, implying that the lower scan rates facilitate an extended period for the anions to retrieve the electrode; this demonstrates the typical capacitive process. In addition, when the scan rates increased, the oxidation sweeps moved more positively, whereas the reduction sweep shifted to a more negative side, and the peak parting increased. This signifies the gradual decrease of the electron movement rate and the loss of reversibility of the electrochemical response, which might be caused by the growing charge diffusion separation with the electrode material at higher scan rates. Nevertheless, there was a slight change in the profile across the peaks for the 3D-NF-1MH sample (Figure 4d), which might be attributed to the various reaction activities of the electrolyte ions following the surface morphology of the sample. The anodic and cathodic sweeps moved in the direction of the negative and positive sides with an increasing scan rate. Figure 3c also shows that the 3D-NF-1MH electrode reveals a leading closed area under the CV or redox current, which is followed by the 3D-NF and 3D-NF-etOH electrodes. Moreover, the enhancement of the capacitive performance of 3D-NF-1MH was also demonstrated by the longer charge/discharge time. The capacitance mostly results from pseudocapacitance, which is in good agreement with the CV observations. The charge/discharge time of the washed electrode was also high compared to the 3D-NF-C electrode, which indicates the effect of washing on the capacitance performance.

Furthermore, for the 3D-NF-3MH sample covered with nanosheet surfaces, a series of CV measurements are shown in Figure 4d at various scan rates within the same potential window. Each of the CV curves is different from the rest of the electrode and shows both Faradic reaction behaviors. As stated, earlier, a high capacitance was acquired from Faradic responses. Upon increasing the scan rate, the reduction peak through the anodic or discharge route moved significantly toward a lower potential; this suggests that the chemical reaction between the formed nanosheets and the electrolyte occurs at a better charge storage for faster scan rates. The opposite trends through the cathodic peaks or charging at higher potentials could also be noticed from the scan of the CV analysis. In the cathodic process, the Faradic reactions nearly faded at higher scan rates because sufficient time was not permitted for the Faradaic reversible reaction. 

The differences in the SC performance of the NF before and after washing with different solvents were further understood by electrochemical impedance spectroscopy (EIS) analysis, as shown in Figure 6 and Figures S1–S5. EIS assessment was also performed to examine the ability of the electrode materials in SC applications. In general, the intercept of the Nyquist profile at the elevated frequency zone signifies the corresponding series resistance (R_s_) of the electrode; this is derived from the inner resistance of the electrode, electrolyte resistance, and resistances of all associates during the reaction process. Every sweep comprises a semicircle arc at elevated frequencies and a straight line in the low-frequency zone. The first semicircle at high frequencies is related to the resistance (R_int_), whereas the semicircle at intermediate frequencies represents the charge transfer resistance (R_ct_). EIS analysis of all samples was performed before and after testing the electrode to assess the electrochemical kinetics behavior; the commercial NF electrode was also analyzed for comparison purposes. The resistance value for the 3D-NF-3MH electrode obtained through the fitted equivalent circuit (Figure S1) was lower (0.4454 ohm) than the 3D-NF-C (0.6949 ohm), 3D-NF-etOH (0.5504 ohm), and 3D-NF-1MH (0.6207 ohm) which has also supported the electrochemical studies. 

From the Nyquist profiles that are displayed in Figure 6, each profile comprises two semicircles and a line. All the samples exhibited different electrochemical kinetics before and after the electrochemical process. In the case of bare Ni foam, there was no change in EIS before and after the electrochemical procedure (CV and CD). The intercept of the semicircle on the real axis at high frequencies is R_s_, which is lower (0.4454 ohm) for the composite than the 3D-NF-3MH electrode; this confirms the good conductivity of the electrolyte and the very low internal resistance of the composite electrode, which may be due to the nanosheet growth on the nickel network structure and the higher electrical conductivity. All these factors might enable the effective contact of electrolyte ions onto the surface and cut the diffusion pathways. In addition, the high frequency semicircle is mainly due to the resistance versus the charge transfer process between the surface of the electrode and the electrolyte. The straight up line, which is equivalent to the imaginary alignment in the low frequency area of the electrodes, indicates the ideal capacitive performance of the samples. Figure 6 suggests that after the electrochemical trial, R_s_ and R_ct_ relics are nearly constant and the steeper slope of the plot in the low-frequency area (which is attained after the procedure compared to that before the electrochemical test for the modified electrodes) indicates the extra noticeable pseudo-capacitive performance of the electrode after the test.

These results indicate that the 3D-NF-3MH electrode has excellent charge storage properties, which might be due to the pore structure and good conductivity of the nickel framework; this framework provides additional surface area and path for charge storage and distribution. Moreover, the nanosized porous structure of the nanosheets might ensure sufficient ions to interact with the active sites in a short time and a great consumption of the electrode; thereby leading to improved capacitance performance.

This study proposes that the washing effect improves substantially the external surface of NF by interacting with the electrolyte and efficiently producing cavities in the nickel framework. These results revealed that this strategy of washing with different solvents could enhance the implementation of NF with the support of a conductive network and different morphologies.

## 4. Conclusions

Free-standing 3D NF is directly used as an efficient SC electrode after chemical/electrochemical modifications. However, most of the treatments require complex reaction procedures. In this study, a facile and efficient washing method was proposed using different concentrations of hydrochloric acid and ethanol to directly influence the surface characteristics of NF with regard to physical properties and electrochemical behavior. Additionally, a comparison of the surface morphology and microstructure characterizations of the bare NF, 3D-NF-etOH, 3D-NF-1MH, and 3D-NF-3MH samples was made. The results demonstrate that washing with hydrochloric acid affects the NF surface, and specifically, the formation of nanosheets/nanoflakes after washing with 3M concentration, which increases the specific surface area that would enhance the specific capacity. 

SC features including cyclic voltammetry, galvanostatic charge/discharge, and impedance characteristics were applied to assess the electrochemical performance of bare and treated NF as free current collectors in a SC application. The comparative electrochemical analysis revealed a better capacitive response of the NF washed with acid (0.77 F/cm^2^) and ethanol (0.1 F/cm^2^). The maximum charge storage of the 3D-NF-3MH electrode was attained after the NF was washed in 3M hydrochloric acid (0.77 F/cm^2^). The key washing effect is the higher specific surface area of NF resulting from densely covered nanosheet growth, which is primarily responsible for obtaining optimum capacitance values. In addition, washing of NF with different acidic concentrations involves the enhancement of a different surface induction method. The application of a washing approach to improve the capacitive behavior of electrodes that is based on 3D NF paves the way for important advancements in electrode technology. Further, as this method is efficient and does not involve complex and expensive process protocols, it might allow the improvement of the SC performance for a wide range of other electrodes that are based on NF.

## Data Availability

Data available in a publicly accessible repository.

## Supplementary Materials

The following are available online at https://www.mdpi.com/article/10.3390/nano11061596/s1, Figures S1–S5: fitted equivalent circuit and EIS fitted graphs.
