# Supplementary material for: Effect of Washing on the Electrochemical Performance of a Three-Dimensional Current Collector for Energy Storage Applications
Source: Nanomaterials (Basel). 2021 Jun 17;11(6):1596. doi: 10.3390/nano11061596 (PMC8234956; doi:10.3390/nano11061596)
Supplement: Supplementary file 1 [file nanomaterials-11-01596-s001.zip › nanomaterials-1259286-supplementary.pdf]

Electronic Supplementary Information

Effect of Washing on the Electrochemical Performance of a Three-Dimensional Current Collector for Energy Storage Applications

Sajid Ali Ansari [1,*], Nazish Parveen [2], Mohd Al Saleh Al-Othoum [1] and Mohammad Omaish Ansari [3]

1 Department of Physics, College of Science, King Faisal University, P.O. Box 400, Hofuf 31982, Al-Ahsa, Saudi Arabia; malothoum@kfu.edu.sa
2 Department of Chemistry, College of Science, King Faisal University, P.O. Box 380, Hofuf 31982, Al-Ahsa, Saudi Arabia; nislam@kfu.edu.sa
3 Center of Nanotechnology, King Abdulaziz University, Jeddah 21589, Saudi Arabia; moansari@kau.edu.sa
* Correspondence: sansari@kfu.edu.sa; Tel.: +966-13-589-9598

The fitted equivalent circuit

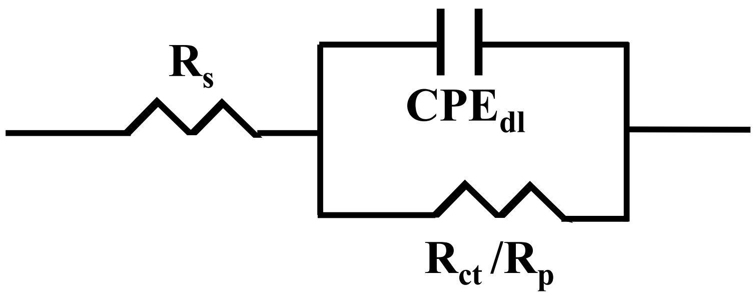

Figure S1. The fitted equivalent circuit of the fabricated electrode.

EIS fitted graph of the 3D-NF-C

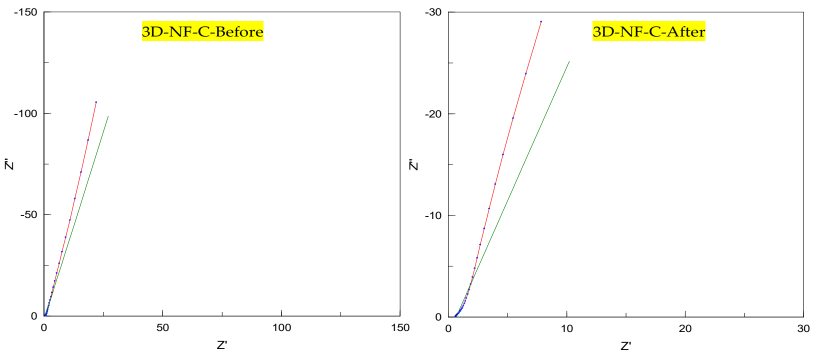

Figure S2. EIS fitted graph of the 3D-NF-C.

EIS fitted graph of the 3D-NF-etOH

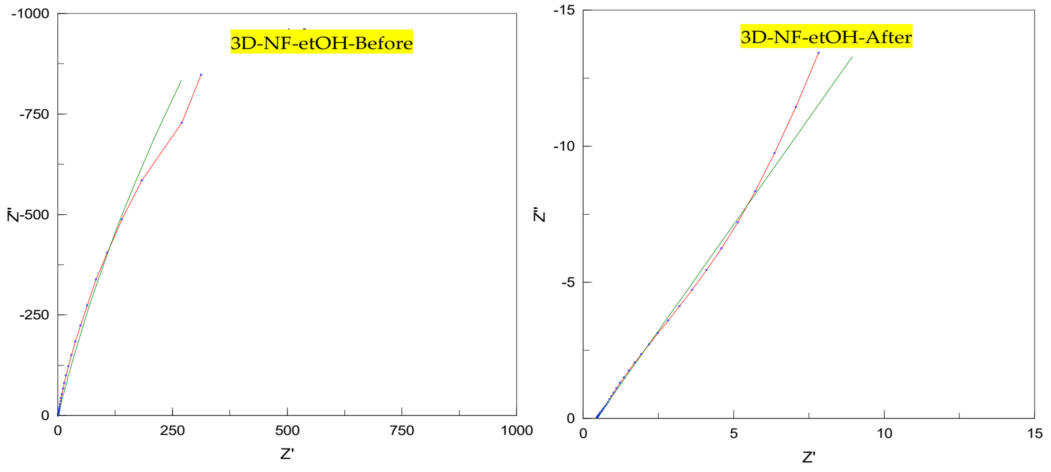

Figure S3. EIS fitted graph of the 3D-NF-etOH.

EIS fitted graph of the 3D-NF-1MH

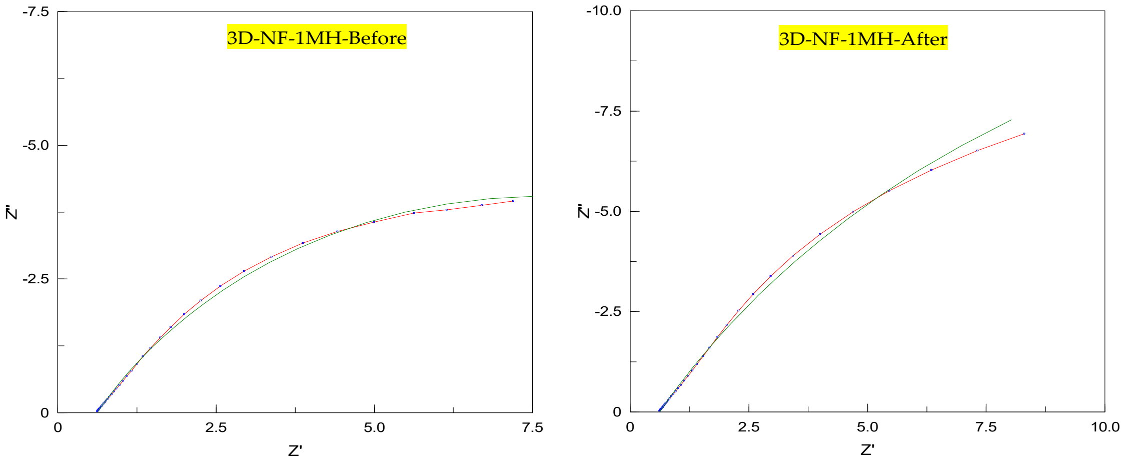

Figure S4. EIS fitted graph of the 3D-NF-1MH.

EIS fitted graph of the 3D-NF-3MH

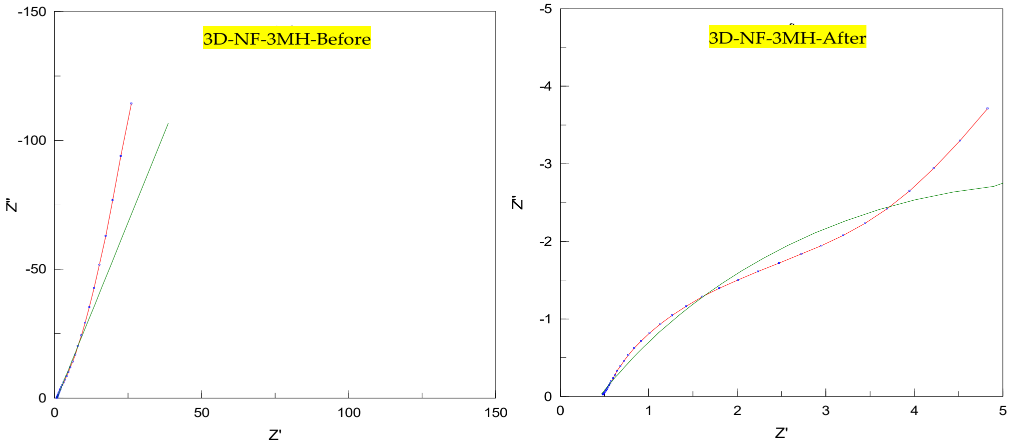

Figure S5. EIS fitted graph of the 3D-NF-3MH.
